# Unified modeling of Familial Mediterranean Fever and Cryopyrin Associated Periodic Syndromes

**DOI:** 10.1186/1546-0096-13-S1-O43

**Published:** 2015-09-28

**Authors:** A Gul, Y Bozkurt, A Demir, B Erman

**Affiliations:** 1 Istanbul University, Istanbul Faculty of Medicine, Department of Internal Medicine, Istanbul, Turkey; 2 Koc University, Computational and Quantitative Biology Lab, Istanbul, Turkey

## Background

Familial Mediterranean Fever (FMF) and Cryopyrin Associated Periodic Syndromes (CAPS) are two prototypical hereditary autoinflammatory diseases, characterized by recurrent episodes of fever and inflammation as a result of mutations in MEFV and NLRP3 genes encoding Pyrin and Cryopyrin proteins, respectively. Pyrin and Cryopyrin play key roles in the multiprotein inflammasome complex assembly, which regulates activity of an enzyme, Caspase 1, and its target cytokine, IL-1 beta. Overproduction of IL-1 beta by Caspase 1 is the main cause of episodic fever and inflammatory findings in FMF and CAPS.

## Objectives

Quantitative models have been used in order to understand how the immune cells and key molecular players interact with each other to constitute the total activity of the immune system. This study aimed to develop a mathematical model to describe the quantitative behavior of all immune cells and inflammatory molecules involved.

## Methods

Critical components involved in the pathogenesis of FMF and CAPS were determined by extensive literature review. A unified dynamical model that captures key aspects of FMF and CAPS was introduced in the form of coupled nonlinear ordinary differential equations. Detailed bifurcation analyses were performed on the model, and simulation results were obtained.

## Results

The model is composed of two subsystems. One of the subsystems, which contains a coupled positive-negative feedback motif, captures the dynamics of inflammation formation and regulation. A comprehensive bifurcation analysis of the model shows that it exhibits three modes, capturing the Healthy, FMF and CAPS cases. The mutations in Pyrin and Cryopyrin are reflected in the values of three parameters in the model. According to the bifurcation analyses performed and simulation results obtained from the model, Caspase 1 level is the most critical parameter in determining the three modes that the model exhibits, “Healthy”, “FMF” and “CAPS”. In accordance with the clinical literature, FMF comes out as trigger-dependent condition, while CAPS is mostly due to autonomous events, mainly associated with self dynamics of the immune response-related inflammasome and associated proteins’ concentrations. In the presence of a trigger, there was a normal increase in inflammation initiators in the Healthy mode. In FMF, the response to trigger introduction is more intense and a severe inflammatory cascade is activated. In CAPS, on the other hand, even in the absence of a trigger, periodically recurring, severe inflammatory episodes are observed. The proposed model also explains why a proCaspase 1 inhibitors can be effective in treating FMF, but not CAPS, for which drugs that directly inhibit IL-1 are used.

## Conclusions

This provisional mathematical model for FMF and CAPS may help explain their pathogenesis and observed clinical behavior as well as designing custom drug therapies for better control of disease activity. Further studies are necessary to link and fine tune all model variables and parameter values to clinical observations and measurements from in vitro experiments, as well as to assess this models potential in the drug development process.

